# Limitations of inflammatory biomarkers in differentiating patients with fungal pneumonia and bacterial co-infection

**DOI:** 10.22034/cmm.2025.345248.1602

**Published:** 2025-08-26

**Authors:** Sergey A. Pogodin, Daniel F. M. Gonzalez, Rajkumar Rajendran, Juan U. Rojo

**Affiliations:** Department of Clinical Laboratory Sciences, School of Health Professions, University of Texas Medical Branch, Galveston, Texas, USA

**Keywords:** Bacterial co-infection, Fungal pneumonia, Inflammatory biomarkers

## Abstract

**Background and Purpose:**

With the increasing number of fungal infections due to antibiotic use and growing numbers of immunocompromised patients, it has become imperative to understand the nature of these infections. Fungal infections, however, remain largely understudied and underdiagnosed, especially when it comes to co-infections with other organisms. This study aimed to compare inflammatory biomarkers in fungal pneumonia patients with and without bacterial co-infections and analyze the frequencies of the causative bacterial and fungal organisms.

**Materials and Methods:**

This retrospective study used electronic medical records from patients diagnosed with fungal pneumonia from January 2013 to December 2023. Fungal and bacterial etiologies were identified with International Classification of Diseases-10 codes and summarized with descriptive statistics. Baseline characteristics, comorbidities, and length of stay were analyzed using descriptive statistics and the Chi-squared test. Inflammatory biomarkers, including C-reactive protein, erythrocyte sedimentation rate, procalcitonin, white blood cell count, body temperature, ferritin, and electrolytes, were collected using Current Procedural Terminology codes and compared using Chi-squared test between patients diagnosed with fungal pneumonia alone or with fungal pneumonia and bacterial co-infection.

**Results:**

A total of 1,024 patients were diagnosed with fungal pneumonia. The most common diagnosis among the patients was unspecified mycosis. Moreover, the most common organism-specific mycotic disease was pneumocystosis. Comorbidities, including diabetes and chronic inflammatory disease, were more common in patients with co-infection (*p* < 0.05). None of the inflammatory biomarkers investigated were statistically significant.

**Conclusion:**

Lack of specificity of most fungal organisms responsible for pneumonia highlights the critical lack of specific diagnostic methods for fungal diseases. The results show that inflammatory biomarkers are not significantly different between fungal pneumonia patients with and without bacterial co-infections.

## Introduction

Fungi are a large and varied group of organisms that play multiple roles in both health and disease [ 1 ]. Billions of fungal infection cases are reported annually, the majority of which are relatively mild, consisting mostly of localized infections of the skin, hair, and nails. However, there are millions of other cases where the fungal infections are severe [ 2 ]. 

One of the major concerns is fungal pneumonia, which is caused as the result of inhalation of fungal spores that germinate in the warm and moist conditions of the lungs. Patients with fungal pneumonia can exhibit flu-like symptoms, such as fever, cough, chest pain, viscous mucus, and joint pain. If the fungal organism enters the bloodstream, it can disseminate and affect the blood, organs, and central nervous system. Furthermore, if the fungal microorganism crosses the blood-brain barrier, the infection can lead to fungal meningitis, which, if left untreated, can be fatal. Fungal infections can be a serious problem for patients with weak or dysfunctional immune systems, mainly among individuals with human immunodeficiency virus (HIV)/acquired immunodeficiency syndrome (AIDS), cancer patients, and individuals undergoing immunosuppressive therapy for bone marrow or stem cell transplantation [ 3 ]. 

In addition, fungal infections are considered emerging diseases [ 4 ]. There has been an upward trend in fungal pulmonary infections between 2013 and 2019 in hospitalized patients between the ages of 14 and 30 years. This trend is stated to be the result of increased number of immunocompromised patients and increased use of antibiotics [ 5 ]. Antifungals, such as azoles, polyenes, echinocandins, and allylamines are the drugs of choice for treating pulmonary fungal infections; however, as their use increases, fungal organisms can develop resistance to antifungal medications by constantly evolving their genome [ 6 ]. Despite the apparent clinical significance of fungal infections, they remain largely understudied and underdiagnosed, compared to other infectious diseases, in part due to a lack of published literature [ 1 ]. As a result of this lack of knowledge, diagnosis and treatment can be delayed, negatively impacting patient outcomes. 

Patients with fungal pulmonary infections are also susceptible to bacterial co-infections. Presence of co-infections can worsen the conditions of patients. To date, little knowledge exists regarding the prevalence of fungal and bacterial co-infections with the most common etiologies involved remaining largely unidentified. Possible combinations of fungal and bacterial co-infections make it difficult to study their potential associations [ 7 ]. Subsequently, few studies have examined the incidence of these co-infections, and the species involved [ 8 ].

Similarly, information regarding diagnostic markers for co-infections is unexplored as most studies analyzing inflammatory biomarkers among patients with co-infections have focused on viral co-infections. For example, interleukin-6, procalcitonin (PCT), and C-reactive protein (CRP) levels were measured in Vietnamese children with respiratory syncytial virus (RSV) alone and in those co-infected with RSV and bacteria. Their results demonstrated significant increases for all three biomarkers, especially PCT, in RSV patients with bacterial co-infection, compared to RSV patients without co-infection. The PCT concentration can be useful in diagnosing and distinguishing children with RSV alone and those with RSV and bacterial co-infection [ 9 ]. 

Another study investigated pro-inflammatory markers associated with fungal and bacterial co-infections. In the aforementioned study, pulmonary computerized tomography clinical manifestations were worse in patients with both fungal and bacterial co-infections, compared to those with fungal infections alone. Moreover, 40.3% of patients with fungal pulmonary infections had bacterial-fungal co-infections, with evidence suggesting a positive correlation between interspecies bacterial and fungal interaction [ 8 ]. Another study found that interleukins were abnormally high in aplastic anemia patients with bacterial or fungal co-infections, compared to patients with aplastic anemia alone [ 10 ]. These findings can also be representative of bacterial co-infections in fungal pneumonia patients and provide important diagnostic relevance.

Therefore, more research is still needed to investigate the role and patterns of inflammatory markers in bacterial and fungal co-infections. The present study analyzed the fungal and bacterial etiologies and the distribution of these pathogens in patients diagnosed with fungal pneumonia. Baseline characteristics, comorbidities, and length of stay (LOS) of patients with fungal pneumonia and bacterial co-infection were analyzed. Moreover, differences in laboratory values for inflammatory markers (CRP, erythrocyte sedimentation rate [ESR], PCT, white blood cell [WBC] count, body temperature, ferritin, and electrolytes) were compared between these patients. Determination of the frequency of fungal and bacterial etiologies can help better understand the prevalence of bacterial organisms in fungal pneumonia patients. This study hypothesized that patients with fungal pneumonia and a bacterial co-infection experience a more severe disease, resulting in longer hospital stays and elevated inflammatory biomarkers, compared to patients with fungal pneumonia alone.

## Materials and Methods

### Study Design

This non-experimental, retrospective, quantitative study was conducted by retrieving medical records of patients aged 18 years or older diagnosed with fungal pneumonia. The selected subjects of the study were divided into two groups: Group 1 included patients diagnosed with fungal pneumonia alone and Group 2 consisted of patients co-infected with fungal pneumonia and bacteria. It must be mentioned that pregnant patients and patients under the age of 18 were excluded. This study was approved by the Institutional Review Board (IRB 23-0389, approval date 01/16/2024) which determined the study to meet the criteria for exemption and did not require participant consent.

### Data Collection and Data Analysis

Electronic medical records of patients diagnosed with fungal pneumonia, with and without bacterial co-infections, were retrieved through EPIC Software from January 1, 2013, to December 31, 2023. Fungal pneumonia was identified by patients that presented one or more of the following ICD-10 codes as part of their medical record: ICD-10 J16.8 (pneumonia due to other specified infectious organisms), B49 (unspecified mycosis), B48.4 (penicillosis), B38.0 (acute pulmonary coccidioidomycosis), B38.2 (unspecified pulmonary coccidioidomycosis), B45.0 (pulmonary cryptococcosis), B59 (pneumocystosis), and/or B37.1 (pulmonary candidiasis). The criteria used to identify patients with a bacterial co-infection were based on positive bacterial growth on record for the Current Procedural Terminology codes for blood (008300), urine (00843), body fluid (180802), and anaerobic/aerobic cultures (182776) collected within 72 h of admission.

Distribution and etiologies of the fungal and bacterial pathogens were identified for each patient using EPIC and summarized using descriptive statistics. After the organisms were counted and similar entries were merged to avoid duplicates, the organisms were organized using crosstabs. 

Baseline characteristics included age, gender, and race/ethnicity. Comorbidities included diabetes (ICD-10 E08), cardiac diseases (ICD-10 151.9), peripheral vascular diseases (ICD-10 173.9), chronic inflammatory disease (CID) (ICD-10 G61; K50; K51; J44), and HIV ICD-10 B20). The LOS was calculated by subtracting the date of discharge or time of death from the date of admission. Descriptive statistics were used to summarize the baseline characteristics, comorbidities, and LOS of patients followed by the Chi-squared test to determine statistical differences between the groups in terms of comorbidities, age, and LOS. 

To compare the two patient groups in terms of inflammatory biomarkers, data were collected from EPIC by retrieving the following laboratory values within 72 h of admission: CRP, ESR, PCT, WBC count, body temperature, ferritin, and electrolytes. In addition, a priori power analysis was performed using G*Power to determine a sample size. With a significance criterion of α = 0.05 to achieve 0.8 power for detecting a medium effect, a sample size of N = 180 was adequate to test our hypothesis. Chi-squared test was the test of choice to compare the groups in terms of statistical differences in inflammatory biomarkers. A *p* value of < 0.05 was used to determine statistical significance. 

## Results

Unspecified mycosis (ICD-10 B49) was the most common code used to detect fungal infection, with 719 entries out of 1,024 total patients. The second most common diagnosis related to fungal pneumonia was pneumocystosis (ICD-10 B59) with 113 entries followed by acute pulmonary coccidioidomycosis (ICD-10 B38.0) with 15 cases, pulmonary cryptococcosis (ICD-10 B45.0) with 9 cases, pulmonary candidiasis (ICD-10 B37.1) with 8 cases, and unspecified pulmonary coccidioidomycosis (ICD-10 B38.2) with 5 cases. 

**Figure 1:**
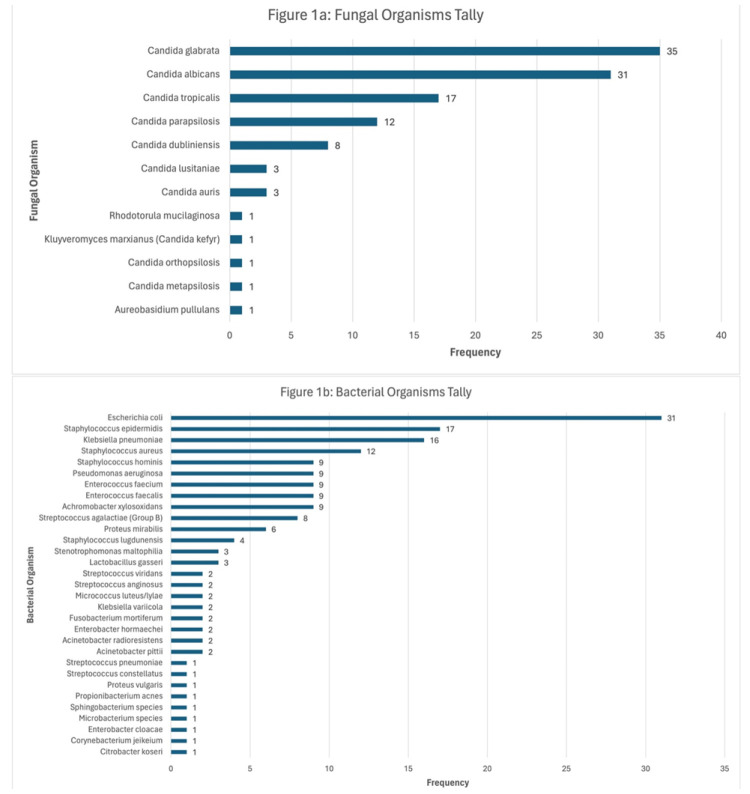
A. *Candida* species were the most common fungal genera isolated from the patient population, with *Candida glabrata* being the most common species with 35 cases. Figure 1. B. The most common bacterial organism isolated from the patient population was *Escherichia coli* with 31 cases, followed by *Staphylococcus epidermidis* with 17 cases, *Klebsiella pneumoniae*, with 16 cases, and *Staphylococcus aureus* with 12 cases. *Staphylococcus hominis*, *Pseudomonas aeruginosa*, *Enterococcus faecium*, *Enterococcus faecalis*, and *Achromobacter xylosoxidans* presented with 9 cases each.

The most common fungal organism isolated from the entire patient population was *Candida glabrata*, with 35 cases, making it the most common isolated overall as well. The four most common fungal organisms following *C. glabrata* were *Candida albicans* with 31 cases, *Candida tropicalis* with 17 cases, *Candida parapsilosis* with 12 cases, and *Candida dubliniensis* with 8 cases. The most common bacterial organism isolated from the population that presented bacterial co-infection was *Escherichia coli*, with 31 cases. The nine most common bacterial organisms following *E. coli* were *Staphylococcus epidermidis* with 17 cases, *Klebsiella pneumoniae* with 16 cases, *Staphylococcus aureus* with 12 cases, *Staphylococcus hominis*, *Pseudomonas aeruginosa*, *Enterococcus faecium*, *Enterococcus faecalis*, and *Achromobacter xylosoxidans* with 9 cases each, and *Streptococcus agalactiae* with 8 cases (Figure 1).

The most common fungal organism isolated from urine cultures was *Candida albicans* with 8 cases. The most common fungal organism isolated from blood cultures was *Candida glabrata* with 29 cases, followed by *Candida albicans* with 23 cases, and *Candida parapsilosis* and *Candida tropicalis* with 11 cases each (Table 1).

**Table 1:** Fungal organism frequencies by culture in patients with and without bacterial co-infections.

Urine Culture Key			
Fungal Organisms	No Co-infection	Co-infection	Total
*Candida albicans*	1	7	8
*Candida glabrata*	0	2	2
*Candida tropicalis*	1	4	5
*Kluyveromyces marxianus (Candida kefyr)*	0	1	1
*Candida dubliniensis*	0	1	1
Blood Culture Key			
**Fungal Organisms**	No Co-infection	Co-infection	Total
*Aureobasidium pullulans*	0	1	1
*Candia glabrata*	9	20	29
*Candida albicans*	8	15	23
*Candida auris*	1	2	3
*Candida dubliniensis*	4	3	7
*Candida lusitaniae*	0	3	3
*Candida metapsilosis*	0	1	1
*Candida orthopsilosis*	1	0	1
*Candida parapsilosis*	9	2	11
*Candida tropicalis*	10	1	11
*Rhodotorula mucilaginosa*	0	1	1
Body Fluid Culture Key			
**Fungal Organisms**	No Co-infection	Co-infection	Total
*Candida glabrata*	2	2	4
*Candida parapsilosis*	1	0	1
*Candida tropicalis*	1	0	1

**Figure 2:**
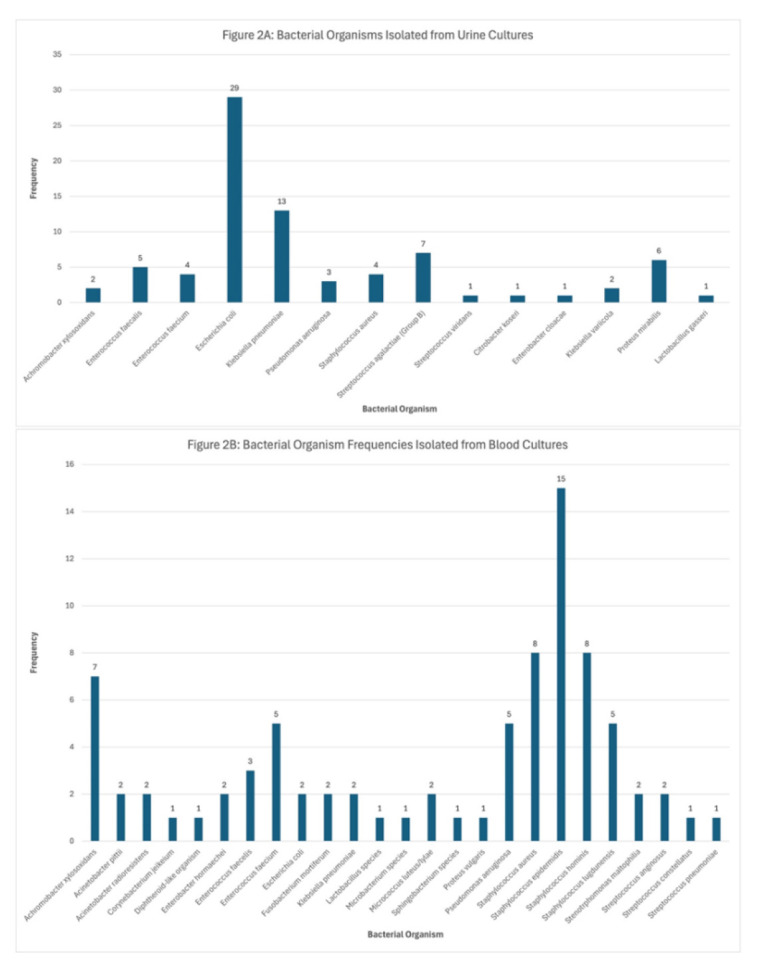
A. The most common bacterial organism isolated from urine cultures was *Escherichia coli* with 29 cases, followed by *Klebsiella pneumoniae* with 13 cases. Figure 2. B. The most common bacterial organism isolated from blood cultures was *Staphylococcus epidermidis* with 15 cases followed by *Staphylococcus aureus* and *Staphylococcus hominis* with 8 cases each and *Achromobacter xylosoxidans* with 7 cases.

As for cultured bacterial microorganisms, the most common bacterial organism isolated from urine cultures was *E. coli* with 29 cases, followed by *K. pneumoniae* with 13 cases. The most common bacterial organism isolated from blood cultures was *S. epidermidis* with 15 cases (Figure 2). 

The highest frequency for race in the study population was Caucasian/White with 787 patients (76.9%), followed by Black/African American with 209 patients (20.4%). The highest frequency for ethnicity in the study population was non-Hispanic or non-Latino with 760 patients (74.2%), followed by Hispanic or Latino, with 236 patients (23%). There were more females than males in the study population, with 573 female patients, respectively (56%). Mean age of the entire study population was 55 years, with a standard deviation of 18.035 (Table 2). 

**Table 2:** Baseline characteristics of the patient population.

Race	Frequency	Percent (%)
Caucasian/White	787	76.9
Black or African American	209	20.4
Asian	18	1.8
Native Hawaiian or other Pacific Islander	2	0.2
American Indian or Alaskan	6	0.6
Unknown	2	0.2
Total	1024	100
**Ethnicity**	Frequency	Percent
Hispanic or Latino	236	23
Non-Hispanic or non-Latino	760	74.2
Patient refused	1	0.1
Unknown	27	2.6
Total	1024	100
**Gender**	Frequency	Percent
Male	451	44
Female	573	56
Age	Mean	SD
	54.83	18.035

**Figure 3:**
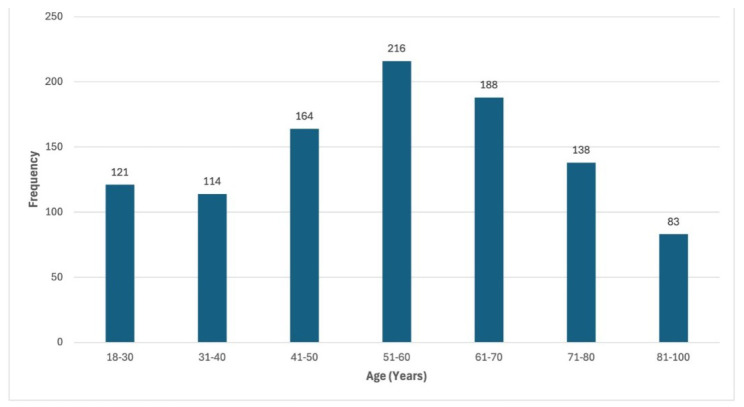
Age range of the study population had a bell curved Gaussian distribution with the mean age between 51 and 60 years.

The age range of the patient population had a bell-curve Gaussian distribution (Figure 3).

The mean and standard deviation for age among patients with fungal pneumonia with or without co-infection was 58.1 + 17.369 and 51.52 + 18.111 years, respectively (Table 3). Mean age of patients with co-infection was higher than that of patients without co-infection. A *p* value of < 0.05 indicates that age is statistically significant.

**Table 3:** Comorbidities, age, and length of stay in patients with and without bacterial co-infections.

Test Variable	No Co-infection	Co-infection	Two-sided *p* value
Patients	508	516
Age (mean + SD)	51.52 + 18.111	58.1 + 17.369	<0.001
Diabetes	5	40	<0.001
CID	32	56	0.009
HIV	55	28	0.002
LOS	17.96 + 33.854	16.95 + 22.034	0.757

CID: chronic inflammatory disease HIV: human immunodeficiency virus, LOS: length of stay

**Figure 4:**
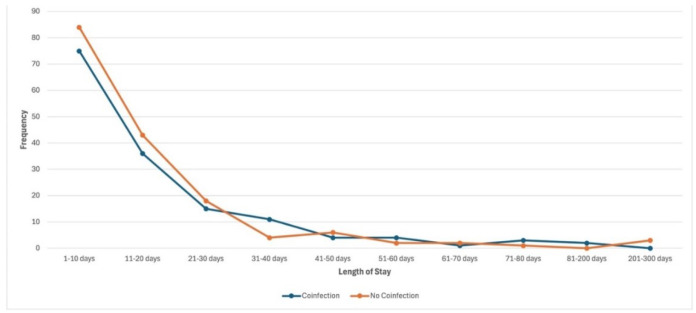
Length of stay had the most variability in the first four data points. Across hospital stays of 1-10 days, 11-20 days, and 21-30 days, there were more patients without co-infections, compared to patients with co-infections. At 31-40 days, there were more patients with co-infections than patients without co-infections. Beyond this point, the group with the higher frequency alternated inconsistently across the remaining intervals.

The LOS for patients with and without co-infection was roughly equivalent (Figure 4). The mean values and standard deviations for patients with and without co-infection were 16.95 + 22.034 and 17.96 + 33.854, respectively. Given the means and a *p* value of 0.757, LOS is likely not a statistically significant variable in regard to patients with and without co-infection. 

When assessing the incidence of comorbidities in the studied population, the total numbers of patients with and without co-infection were 516 and 508 cases, respectively. For patients with comorbidities with and without co-infection, the numbers of cases were 40 and 5 for diabetes, 56 and 32 for CID, and 28 and 55 for HIV, respectively (Table 3). The *p* values of diabetes, CID, and HIV were < 0.001, 0.009, and 0.002, respectively, which indicate that the variables were statistically significant. 

The most common diagnosis alongside HIV was pneumocystosis, with 69 out of the 83 total HIV cases followed by unspecified mycosis, which accounted for 9 cases. For diabetic patients, unspecified mycosis was the most common diagnosis with 41 out of 45 cases. 

To evaluate if laboratory markers correlated with a bacterial co-infection, inflammatory biomarker values were evaluated for patients with and without co-infection. From these, C-reactive protein levels were analyzed in six patients, including two patients with co-infection and four without co-infection. Mean values of CRP were 0.67 mg/dL + 0.89 and 2.36 mg/dL + 4.16 for patients with and without co-infection, respectively. An independent samples t-test gave a two-sided *p* value of 0.619 which indicated no statistical significance between the two groups. Similarly, ESR, PCT, WBC count, body temperature, and ferritin were evaluated, and none demonstrated a statistically significant difference between the two groups. There was no statistically significant difference in electrolyte values between patients with fungal pneumonia, with or without bacterial co-infection. The results are summarized in Table 4. 

**Table 4:** Inflammation biomarker values in patients with and without bacterial co-infections.

Inflammation Biomarker	Mean + SD (No Co-infection)	N	Mean + SD (Co-infection)	N	Two-sided *p* value
CRP (mg/dL)	2.36 + 4.16	4	0.67 + 0.89	2	0.619
ESR (mm/hr)	54.31 + 40.25	13	65.81 + 37.31	26	0.382
PCT (ng/mL)	2.06 + 5.31	37	1.45 + 3.38	35	0.567
WBC Count (cells/μL)	2267.83 + 8427.23	23	5513.72 + 15353.84	16	0.401
Body Temperature (F)	97.87 + 0.86	423	97.86 + 1.0	379	0.836
Ferritin (ng/mL)	731.5 + 768.97	23	648.28 + 757.02	24	0.71
Sodium (mmol/L)	137.21 + 4.62	184	137.07 + 3.9	190	0.738
Potassium (mmol/L)	4.14 + 0.52	184	4.18 + 0.45	190	0.506
Chloride (mmol/L)	102.64 + 5.44	184	102.94 + 5.30	190	0.598
CO_2_ (mmol/L)	25.5 + 3.73	180	25.27 + 4.35	188	0.592

CRP: C-reactive protein, ESR: erythrocyte sedimentation rate, PCT: procalcitonin, WBC: white blood cell

## Discussion

The striking majority of unspecified mycotic diseases are likely due to a lack of sensitive and specific diagnostic methods and the absence of standardization of ICD-10 codes that can provide detailed information regarding the fungal causative agent. Methods, like chemical testing and fungal culture are time-consuming, and often treatment is initiated before full speciation is made. The consequences of this lack of specificity can lead to delays in diagnosis and, subsequently, delays in treatment [ 11 ]. Without complete speciation, tracking the prevalence of any clinically isolated microorganism can be difficult. Likewise, it can make it difficult to perform antifungal susceptibility testing. *Candida auris* is a rapidly emerging species with multidrug resistance characteristics [ 12 - 14 ]. The patients in the present study only had three identifiable cases of *C. auris*, yet no distinction of drug resistance was specified. Therefore, better methods for fungal diagnosis are needed to detect and identify the pathogenic organism to prevent delays in treatment and subsequent harm to the patient. Hence, it is recommended that laboratory settings report fungal infections at least to the genus level. 

Among the organism-specific mycotic diseases, *Pneumocystis jirovecii* and *Pneumocystis carinii* were the most common diagnoses made as pneumocystosis, which are frequently associated with infections in immunocompromised patients [ 15 ]. This was observed in the study population of the present research as the most common diagnosis for HIV-positive patients was pneumocystosis. *Pneumocystis jirovecii* is a fungal organism that is very commonly associated with HIV, with approximately two-thirds of patients with AIDS being infected with *P. jirovecii* during the AIDS epidemic [ 16 ]. Therefore, the findings of the present study support the ongoing research on *P. jirovecii* and *P. carinii* as significant HIV-associated opportunistic pathogens. 

The most common fungal organisms isolated from the various cultures were *Candida* species, which was expected, given that they are the most common fungal infection genus in the United States [ 17 ]. Other fungal genera responsible for systemic and opportunistic infections, such as *Talaromyces* and *Aspergillus*, are uncommon, which corroborates the lack of diversity in fungal organisms isolated from the study population. The most common fungal organisms isolated from urine and blood cultures were *C. albicans* and *C. glabrata*, respectively. This finding is supported by the literature [ 18 , 19 ].

The most common bacterial organisms isolated from urine and blood cultures were *E. coli* and *S. epidermidis* respectively. It was expected that the most common bacterial organism isolated from urine cultures would be *E. coli*. The results are in line with previous research, which indicates that the most common organism responsible for urinary tract infections is *E. coli* [ 20 ]. *Staphylococcus epidermidis* is the most common bacterial organism isolated from blood cultures due to the nature of the organism being a common contaminant or true bacteremia [ 21 ]. Regarding blood culture contamination, *S. epidermidis* is normal skin flora and can contaminate the blood culture bottle if the blood collection site is not properly cleaned [ 22 ]. The second most common bacterial organisms isolated from blood cultures were *Staphylococcus hominis* and *Staphylococcus aureus* with eight cases each. *Staphylococcus hominis* is a common commensal organism found on human skin and likely represents a blood culture contaminant similar to *S. epidermidis*. The organism most commonly associated with the blood infection pathogen is *S. aureus*. Patients with *S. aureus* skin lesions are at increased risk of the organism spreading to the bloodstream if they are infants, elderly, or patients with additional comorbidities, such as heart disease, diabetes, renal diseases, or HIV infection [ 23 ]. 

It is noteworthy that there were more females than males in the study population as well as in the co-infection group, compared to the no co-infection group. Since *E. coli* was the most frequent bacterial organism isolated from urine cultures, the most likely reason for this increase in female patients in the co-infection group is that females are more prone to urinary tract infections, compared to males [ 24 ].

Mean age of the study population was 55 years, which was expected given that middle-aged and older patients are more susceptible to infections due to age-related decline in the function of the immune system [ 25 ]. Mean age of patients with co-infection was 7 years higher than that of the patients without co-infection. Additionally, a two-sided *p* value of < 0.05 indicated that age is a significant variable regarding the presence of co-infection. Significance of this variable suggests that older patients are at higher risk of developing a co-infection, compared to younger patients, even by several years. Therefore, it may be important to monitor patients within this age range for the presence of co-infection. 

The LOS was not a significant variable when comparing patients with and without co-infection. A plethora of factors, excluding the presence of co-infection, may be responsible for variations in LOS, including comorbidities, response to treatment, age, immune response, or severity of disease. 

Regarding comorbidities, none of the patients presented with peripheral vascular diseases or cardiac diseases. Diabetes was present in the majority of cases in the co-infection group, compared to the group without co-infection. A *p* value of < 0.05 also confirmed the significance of diabetes as a variable. Diabetic patients are more prone to infections, and it is worth noting that they can be afflicted with multiple pathogenic organisms rather than a single pathogenic organism, given the laboratory results [ 26 ]. Although it is unclear which causative organisms were responsible for the fungal pneumonia of the patient, given the unspecific nature of the diagnostic code, it is possible to deduce likely pathogens based on current scientific research.

Diabetes is a metabolic condition characterized by elevated blood glucose levels as a result of impaired or absent insulin secretion and insulin function or both. In the case of diabetic patients, changes in blood sugar levels can impair the phagocytic capabilities of leukocytes, leading to an increased risk of bacterial and fungal infections of the skin and soft tissues [ 27 ]. *Candida* species are the most common causes of fungal infections in diabetic patients, typically manifesting as foot ulcers [ 28 ]. Diabetic foot ulcers account for a 155-fold increased risk of amputation, compared to patients without diabetes; hence, patients must be swiftly treated before the infection spreads and amputation is required [ 29 ]. Unfortunately, the current method for treating diabetic foot ulcers is to identify and treat the bacterial organism responsible while ignoring fungal infections [ 28 ]. The microorganism must be identified and treated regardless of whether it is of bacterial or fungal origin to prevent the condition of the patient from worsening and leading to a preventable amputation. 

Patients with CID were more frequently affected by co-infections, compared to those without CID. This suggests that patients with a chronic inflammatory disease were more likely to experience co-infection, compared to patients without CID. Moreover, HIV was more prevalent in patients without co-infections, compared to those with co-infections. Altogether, patients with diabetes and CID demonstrated a higher rate of bacterial co-infection, compared to those without diabetes and CID. Both diabetes and CID are conditions known to cause tissue damage and impair the immune system, thereby increasing the risk of infection development. Results of the present study support evidence that comorbidities predispose individuals to infections, mainly bacterial co-infections [ 30 ].

None of the assessed inflammatory biomarkers were statistically significant. Variations between bacterial, fungal, and viral infections may have different effects on inflammatory biomarkers either with another organism or comorbidity. Another explanation is that the selected inflammatory biomarkers were less sensitive to lung infection. Likewise, the inflammatory biomarkers assessed in this study are non-specific and thus cannot be used to differentiate causative agents or clinical conditions. The ESR and CRP have limited sensitivity and specificity; therefore, the values analyzed may not be different enough to be used as proxies for identifying patients with fungal pneumonia and bacteria co-infection. Previous studies have found that cytokines are more sensitive to lung infection than CRP, PCT, and body temperature [ 31 ]. These results do not support the original hypothesis of this study which indicated that inflammatory biomarkers would be significantly increased in fungal pneumonia patients with co-infections compared to fungal pneumonia patients without co-infections. 

## Conclusion

This study provided important data regarding the nature of fungal infections in patients with diabetes and HIV as well as frequencies of other bacterial and fungal organisms. Patients with HIV were predominantly diagnosed with pneumocystosis, and diabetics were diagnosed with unspecified mycosis, with the most likely organisms being *Candida* species. Additionally, the lack of specificity of most fungal organisms responsible for pneumonia highlights the critical lack of specific diagnostic methods for fungal diseases. 

Results of this study demonstrated the limitation of routine pro-inflammatory markers (CRP, ESR, PCT, WBC count, body temperature, ferritin, and electrolytes) in differentiating patients with fungal pneumonia with and without bacterial co-infections. Epidemiology varies significantly among bacterial, fungal, and viral infections and may affect inflammatory biomarkers differently when combined with another organism or comorbidity. Experimental observational studies can expand the results of this study, as investigating the relationship between co-infections and inflammatory biomarkers can be better established by measuring additional proinflammatory biomarkers and completing fungal identification. In clinical practice, it would be prudent to screen patients older than 57 years who are present with pneumonia and comorbidities, mainly diabetes and CID, for fungal pathogens and bacterial infections mainly from blood cultures. Likewise, clinical sites should introduce the identification of fungal isolates, at least to the genus level, to improve epidemiological data and better tailor treatment. 
